# Detection of viral contamination in cell lines using ViralCellDetector

**DOI:** 10.3389/fmicb.2025.1595180

**Published:** 2025-08-13

**Authors:** Rama Shankar, Shreya Paithankar, Suchir Gupta, Bin Chen

**Affiliations:** ^1^Department of Pediatrics and Human Development, College of Human Medicine, Michigan State University, Grand Rapids, MI, United States; ^2^Department of Pharmacology and Toxicology, College of Human Medicine, Michigan State University, East Lansing, MI, United States; ^3^Department of Computer Science and Engineering, College of Engineering, Michigan State University, East Lansing, MI, United States

**Keywords:** cell lines, viral contamination, bacterial contamination, differentially expressed genes, RNA-seq data, random forest, machine learning

## Abstract

**Background and aims:**

Cell lines are widely used in biomedical research to investigate various biological processes, including gene expression, cancer progression, and drug responses. However, cross-contamination with bacteria, mycoplasma, and viruses remains a persistent challenge. While the detection of bacterial and mycoplasma contamination is relatively straightforward, identifying viral contamination is more difficult. To address this issue, we developed ViralCellDetector, a tool designed to detect viral contamination by mapping RNA-seq data to a comprehensive viral genome library.

**Methods:**

ViralCellDetector processes RNA-seq data from any host species by first aligning reads to the host reference genome, followed by mapping the unmapped reads to the NCBI viral genome database. Viral presence is determined using stringent criteria based on the number of mapped reads and viral genome coverage. To further enable the detection of viral contamination from unknown sources, we identified host genes that are differentially expressed during viral infection and used these markers to train a machine learning model for classification.

**Results:**

Using ViralCellDetector, we found that approximately 10% (110 samples) of RNA-seq datasets involving MCF7 cells were likely contaminated with viruses. The tool demonstrated high sensitivity in detecting viral sequences. Furthermore, the machine learning model effectively distinguished infected from non-infected samples based on human gene expression profiles, achieving an AUC of 0.91 and an accuracy of 0.93.

**Conclusion:**

Our mapping-based approach enables robust detection of viral contamination in RNA-seq data from any host organism, while the marker-based approach accurately identifies viral infections specifically in human cell lines. This capability can help researchers detect and avoid the use of contaminated cell lines, thereby improving the reliability of experimental outcomes.

## Introduction

Cell lines are invaluable tools in biomedical research as they enable scientists to investigate disease mechanisms and develop new treatments. They are widely used to study a wide range of biological processes, including cell signaling, gene expression, and drug metabolism. Additionally, cell lines play a crucial role in assessing the efficacy and toxicity of new drugs prior to vivo testing. These cell lines can be derived from various sources, such as patient-derived cancer cells, human primary cells, or cells from animals. Immortalized cancer cell lines are often shared across laboratories and research studies. However, contamination with bacteria and mycoplasma is a significant concern (Corral-Vázquez et al., 2017). To mitigate these contamination and ensure experimental integrity, it is essential to follow established best practice guidelines for cell line maintenance and handling (Baust et al., 2017; Reid, 2017).

In addition to bacterial and mycoplasma contamination, viral contamination is a significant concern in cell line-based research, though it is much challenging to detect. Viral contamination can originate from the environment or from the original tissue source used to establish the cell lines. Although human cell lines may potentially harbor latent or active virus, there are limited approaches available for detecting these viruses (Shioda et al., 2018; Cheval et al., 2019; Uphoff et al., 2019; Dolskiy et al., 2020). Many existing approaches are designed to primarily detect only a small subset of well-known pathogenic viruses, such as cytomegalovirus (CMV), Epstein–Barr virus (EBV), human herpesvirus 6 (HHV-6), HHV-7, human polyomavirus BK (BKV), human polyomavirus JC (JCV), human adenovirus (ADV), human parvovirus B19 (B19V), hepatitis B virus (HBV), human T-cell leukemia virus type 1 (HTLV-1), HTLV-2, human immunodeficiency virus 1 (HIV-1), HIV-2, hepatitis A virus (HAV), and hepatitis C virus (HCV). These detection methods, whether PCR-based or reliant on specific viral sequence reads, are tailored for individual viruses, making it challenging to apply them broadly to research scenarios.

Recently, several algorithms have been developed to detect viral integration into the human genome using sequencing data (Kostic et al., 2011; Chen Y. et al., 2013; Li et al., 2013; Naeem et al., 2013; Schelhorn et al., 2013; Wang et al., 2013, 2015; Xia et al., 2019; Selitsky et al., 2020). Among these, VirTect (Xia et al., 2019) is highly advanced and can detect virus integration sites based on whole transcriptome sequencing (RNA-Seq) data. Additionally, an AI-Enabled-Virus-Detect tool (Ghorbani et al., 2024) has been reported to detect viral sequences using a BLAST-based approach. In this method, the authors perform *de novo* assembly of unmapped host reads to identify potential viral contigs. However, this approach presents a limitation, as even a minimal overlap of two reads can lead to contig formation, increasing the likelihood of false-positive viral identifications. Despite these advances, there remains a lack of broadly applicable tools for detecting viral contaminations across various host reference samples. Many existing mapping-based approaches assume that viral sequence reads are abundantly present in sequence data. However, widely adopted polyA-based library preparation protocols primarily enrich for host transcripts with polyadenylated tails, which may limit the detection of viral transcripts. Therefore, there is a critical need for a robust and scalable tool that can detect viral contaminations across diverse sample types using standard RNA-seq data.

To address these limitations, we developed a ViralCellDetector tool that leveraged the ultrafast STAR aligner (Dobin et al., 2013), followed by the BWA aligner, to incorporate all known viruses from the NCBI virus database. Our tool first maps RNA-seq data to any given host reference genome and transcriptome and subsequently maps unmapped reads to a comprehensive viral genome database. To enhance the specificity, users can exclude viruses not affecting their respective host and applied stringent criteria to accurately identify true viruses. As demonstration of the tool’s utility, we analyzed RNA-seq data from more than 1,000 experiments performed on MCF7 cells to identify the potentially infected samples. Additionally, we implemented a machine learning approach using this dataset to identify host gene expression biomarkers associated with viral contamination. This biomarker-based approach exhibits robust performance and is independent of viral species and library preparation protocols. However, it performs best in samples where viral contamination has induced alterations in host cell biology. We anticipate that the integration of both mapping and biomarker-based approaches will empower researchers to effectively identify potential viral contaminations in their cell lines using standard RNA-seq data, thus ensuring the reliability and reproducibility of their experimental results.

## Methods

**Figure 1:**
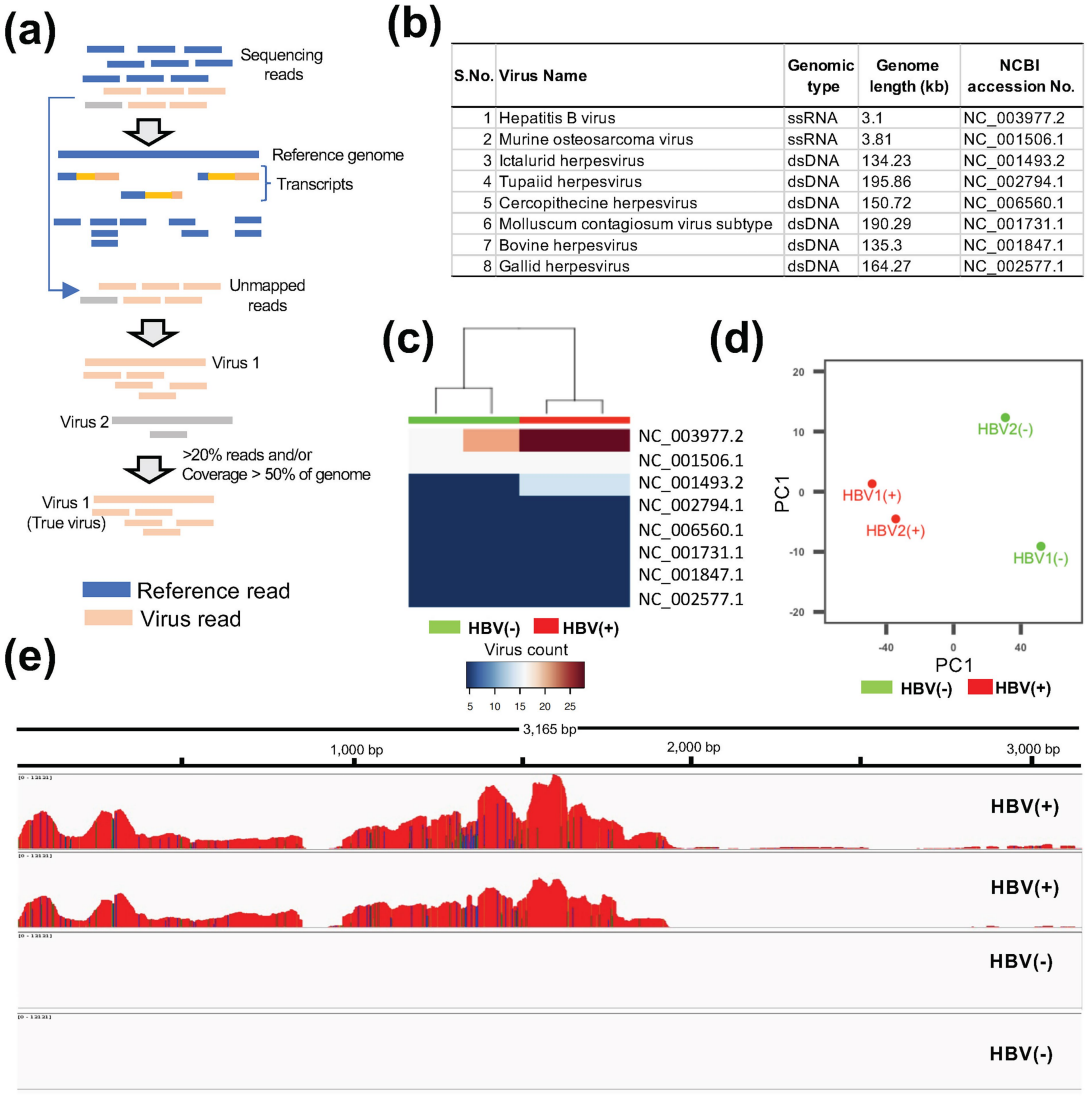
Detection of viral contamination using ViralCellDetector. **(a)** Workflow of the ViralCellDetector pipeline for viral detection. **(b)** List of viruses detected in HBV-positive (HBV+) and control (HBV−) samples. **(c)** Heatmaps illustrating the normalized abundance of viruses in HBV+ and HBV− samples. **(d)** Principal component analysis of HBV+ and HBV− samples. **(e)** Integrated Genomics Viewer (IGV) display of base coverage across the HBV genome in HBV+ and HBV− samples. Multiple tracks represent read alignments for each of the four libraries, mapped to the hepatitis B virus reference genome.

The overall workflow of ViralCellDetector is illustrated in Figure 1a. The fastq files from samples were used as input in ViralCellDetector pipeline. The first step of ViralCellDetector is to align the sequencing reads to the reference genome and its corresponding annotation file (e.g., Hg38 transcriptome with ENSEMBL GRCh38.p3 annotation in case of human cells) using STAR aligner (Dobin et al., 2013). Unmapped reads were subsequently mapped to the viral genome database available from NCBI^1^ using BWA (Li and Durbin, 2009). The advantage of using BWA over STAR aligner for viral genomes is that it can retain alignments even when only one read from a paired end read maps to the viral genome. To reduce false positives, we calculate the total number of reads mapped to each viral genome. Additionally, we estimate the percentage of genome coverage to more accurately identify true viral presence.

### Dataset

We downloaded the SRA sequencing data from GEO^2^. All the sequencing data were further converted into fastq files using the SRAtoolkit^3^. The fastq files were used as input in the ViralCellDetector to detect the putative viruses.

### Data processing

ViralCellDetector is designed to accept raw sequencing data as input. It separates paired end reads into two sets: those mapping to the host reference genome, and those that potentially belonging to viral genomes. Reads aligning to the host genome and transcriptome were first mapped using the ultrafast STAR aligner with default parameters (Dobin et al., 2013). Unmapped reads left after host reference mapping were then aligned to the NCBI viral genome database using the BWA aligner with default parameters (Li and Durbin, 2009). The viral genome sequences can be downloaded from the NCBI virus database (see Footnote 1). After viral sequence download from NCBI, the endogenous retroviruses may be removed before indexing the viral genomes. Once the viral reads were mapped to the viral genome, we applied the following criteria to filter out false positives: (1) at least 20% reads should be mapped to the viral genome (based on our positive sample mapping), (2) the continuous coverage of the viral genome should be more than 50%, and (3) the virus should be known to infect the host.

### RNA-seq analysis

All the sequencing reads were mapped on Hg38 transcriptome using the ENSEMBL GRCh38.p3 annotation with the STAR aligner (Dobin et al., 2013). Gene counts obtained from the alignment were used for identification of differentially expressed (DE) genes. The edgeR package (Robinson et al., 2010) was employed to quantify DE genes based on following criteria: log_2_ fold change ≥1 or ≤−1 and an adjusted *p*-value (False Discover Rate) ≤ 0.01. DE genes were identified between the two groups (control and infection) in two independent datasets. Gene ontology enrichment and KEGG pathways analyses were performed using the enrichR (Chen E. Y. et al., 2013; Kuleshov et al., 2016). DE genes involved in viral infection-related pathways were further utilized as features in machine learning model. Data visualization was conducted using the ggplot2 package in R. All the analyses were performed on R (version 4.2.1).

### Random forest for feature selection and prediction

Random forest (RF) is a robust and widely used machine learning algorithm based on bagging techniques, known for its consistent performance across various classification tasks (Subash et al., 2022; Wadood et al., 2022). It consists of an ensemble of independent decision trees, where the final prediction is determined by aggregating the outputs from all trees (Montes et al., 2021). Key hyperparameters influencing model performance, namely ‘ntree’ (number of trees), ‘mtry’ (number of variables tried at each split), and ‘nodesize’ (minimum size of terminal nodes); can be tuned by the user (Oukawa et al., 2022). In our study, we set ‘ntree’ to 600 and ‘mtry’ to 8 for feature selection and classification. The dataset was randomly partitioned into 80% training and 20% testing subsets. We employed a recursive feature elimination (RFE) to select informative features. To evaluate the stability and generalizability of the model, we implemented 10-fold cross-validation. Features that achieved an accuracy of ≥0.9 across cross-validation folds were retained for final model training and testing. Model performance on the test dataset was assessed using accuracy, area under the curve (AUC), sensitivity, and specificity. Additionally, to address class imbalance, we applied downsampling of the majority class and repeated 10-fold cross-validation to ensure the robustness and reliability of the classifier.

## Results

### Validation of the mapping-based viral contamination detection approach

The workflow of the pipeline is provided in Figure 1a. To validate the tool, we analyzed publicly available RNA-seq datasets containing known viral infection/contamination. One dataset included Hepatitis B virus (HBV) infected samples (GSE65485), comprising two control and two infected samples. The second dataset contained SARS-CoV-2 infected cell lines (GSE187420), consisting of three control and three infected samples. In the HBV dataset, we identified a total of eight viruses in the list (Figure 1b). However, upon examining the expression patterns in the control and infected samples, the HBV-infected samples exhibited the highest abundance of HBV reads compared to control samples (Figure 1c). Furthermore, a clear transcriptional distinction between control and infected samples was observed (Figure 1d). Visualization of read coverage across viral genomes revealed that only the HBV genome achieved more than 90% coverage (Figure 1e), confirming the presence of HBV infection. Similarly, in the SARS-CoV-2 dataset (GSE187420) (Xing et al., 2022), infected samples displayed a high proportion of unmapped reads when aligned to the human reference transcriptome (Supplementary Table S1), along with a substantial number of reads mapping to the SARS-CoV-2 genome (Supplementary Figure S1). These findings support the effectiveness of our pipeline in accurately detecting viral contaminations in RNA-seq data derived from cell lines.

### Comparison with other tools in simulated data

To further evaluate the applicability of the ViralCellDetector tool, we generated six simulated paired-end FASTQ files set by combining varying proportions of viral reads (10, 20, 30, 40, and 50%) from different viruses with host-derived FASTQ reads. We then applied the ViralCellDetector pipeline to these datasets. Additionally, we compared its performance to another method, referred to as the “AI-enabled tool” (Ghorbani et al., 2024). Notably, this tool utilizes *de novo* assembly of unmapped reads from the host genome, followed by virus detection using the Basic Local Alignment Search Tool (BLAST). Despite being labeled as AI-based, the method relies solely on BLAST and does not implement any artificial intelligence algorithms.

**Figure 2:**
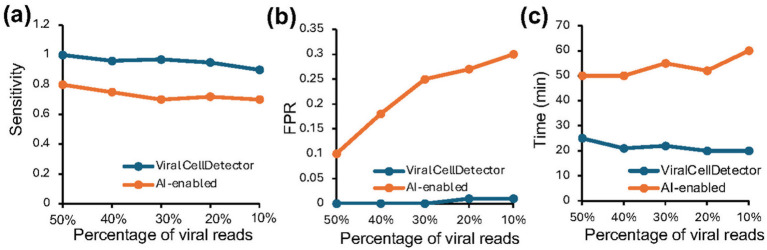
Comparison of ViralCellDetector with other tools on simulated data containing varying proportions of viral reads mixed with host sequences. **(a,b)** Comparison of sensitivity and false positive rate (FPR) between ViralCellDetector and other AI-enabled tools reported to detect viruses in samples. **(c)** Time taken to process the simulated data.

We assessed the performance of ViralCellDetector and the AI-enabled tool by computing sensitivity and false positive rate (FPR) across all simulated datasets. We also recorded the time required by each method to process each sample. ViralCellDetector consistently outperformed the AI-enabled tool in both sensitivity and FPR (Figures 2a,b). Moreover, it completed analyses significantly faster, delivering a putative virus list in much less time (Figure 2c), highlighting the efficiency of the ViralCellDetector tool.

### Detection of viral contamination from unlabeled dataset

**Figure 3:**
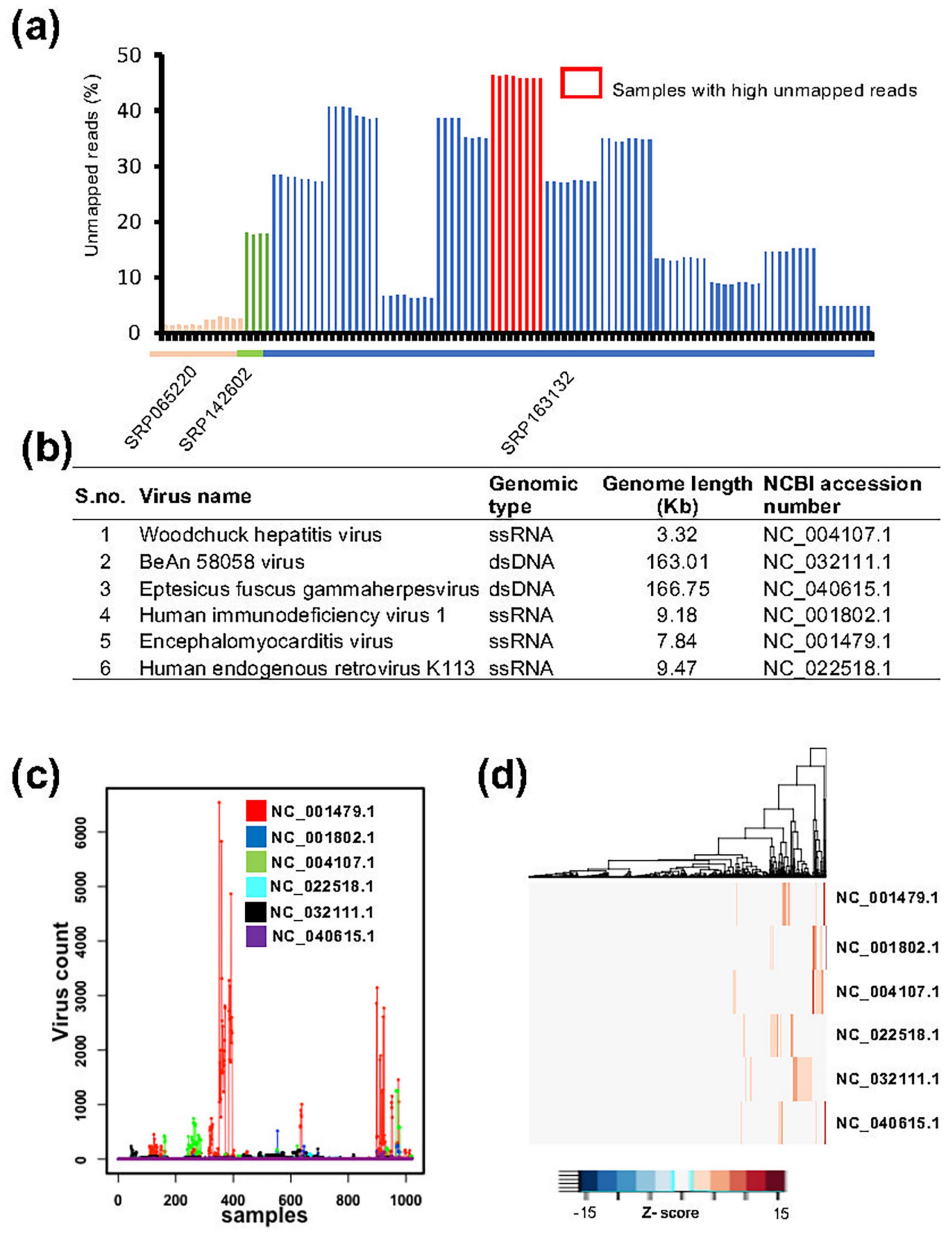
Detection of viral contamination using ViralCellDetector on MCF7 cell line data. **(a)** Percentage of unmapped reads across samples from three independent studies. The highest percentage of unmapped reads (45%) was observed in eight samples from SRP163132 study. These samples were analyzed for viral detection, resulting in the identification of six viruses. **(b)** List of viruses detected in MCF7 samples by ViralCellDetector. **(c)** Expression levels of the six identified viruses across 1,021 MCF experiments, revealing a subset of samples with viral contamination. **(d)** Heatmaps illustrating elevated expression of the six viruses across various MCF7 experiments.

To detect viral contaminations in unlabeled (without known contamination) RNA-seq data, we collected MCF7 cell line datasets from multiple studies (SRP065220, SRP142602, and SRP163132). The sequencing data in these studies were generated using a RiboMinus approach, enabling the capture of all expressed mRNAs from humans and non-human sources, including viruses (Zhao et al., 2018; Chen et al., 2020). Specifically, the SRP065220 dataset includes 12 MCF7 samples, SRP142602 includes four samples, and SRP163132 includes 128 samples. The raw sequencing reads were downloaded and first mapped to the human reference genome and transcriptome. For each sample, the proportion of unmapped reads was then calculated. We observed that eight samples exhibited more than 45% unmapped reads (Figure 3a), suggesting potential viral contaminations. This observation is consistent with previous report (Yuan et al., 2021) and our data (Supplementary Table S1), which demonstrate that viral infections/contaminations are often associated with elevated levels of unmapped reads in RNA-seq data. The unmapped reads were then mapped to the viral genome using ViralCellDetector pipeline, resulting in the detection of a total of 22 viruses (Supplementary Figure S2). After applying stringent criteria, we identified six viruses: three dsDNA viruses (Woodchuck hepatitis virus, BeAN 58,058 virus, and *Eptesicus fuscus* gamma herpes virus) and three ssRNA viruses (Human immunodeficiency virus 1, Encephalomyocarditis virus and Human endogenous retrovirus) (Figure 3b).

To further investigate the presence of these viruses in MCF7 cells, we collected RNA-seq data from over 1,021 MCF7 samples. Raw reads were first mapped to the human reference transcriptome. The unmapped reads of each sample were then analyzed to assess the expression levels of six identified viruses. Across all the samples, we observed considerable variation in the expression levels of these viruses (Supplementary Figure S2). To identify the experiments with exceptionally high viral count, we ranked the viral expression values and applied an upper quartile threshold to select samples with markedly elevated viral counts (Supplementary Figure S3). Using this approach, we identified approximately 110 samples (~10% of total) that exhibited significantly higher viral expression counts compared to the remaining samples (Figures 3c,d), suggesting the likely presence of one or more of these viruses. However, we noted that around 50% of these samples originated from studies (e.g., GSE106694, GSE103520, GSE100099, GSE63189, and GSE67295) in which viruses were intentionally introduced through transfection as part of the experimental design. Therefore, not all detected viral signals necessarily indicate unintended contamination. Furthermore, the presence of viral sequences does not always imply a biological impact on host gene expression. In many cases, the detected viral reads simply reflect the viral load in the samples, without indicating any downstream functional consequences in host cells.

### Identify DE genes involved in viral contamination in different cell lines

**Figure 4:**
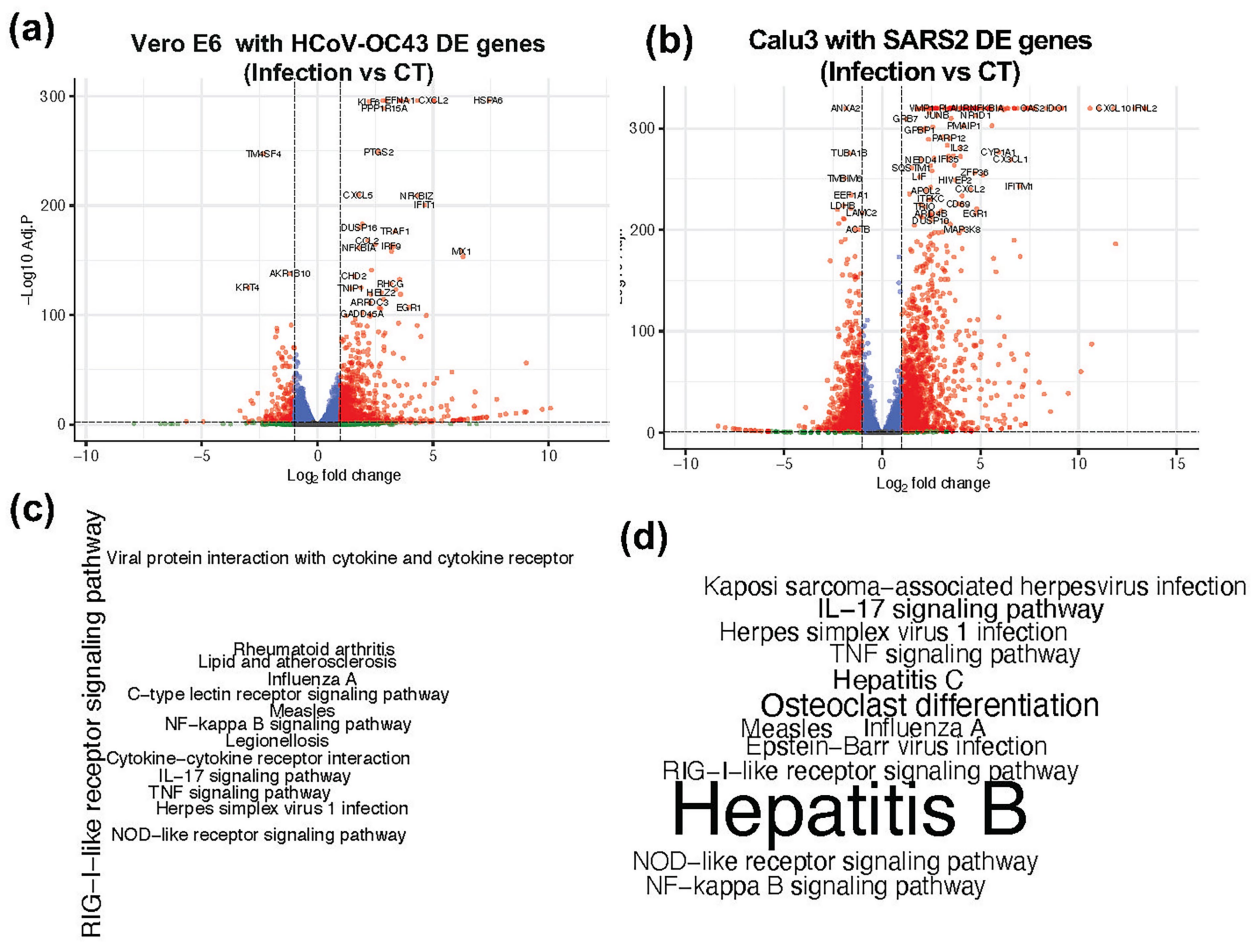
Differentially expressed genes and their KEGG pathway enrichment in two different cell lines. **(a,b)** Volcano plot showing the differentially expressed (DE) genes in Infected vs. Control (CT) samples from **(a)** Vero 6 cells and **(b)** Calu3 cells. **(c,d)** KEGG pathway enrichment analysis of these DE genes in **(c)** Vero 6 cells and **(d)** Calu3 cells, highlighting pathways associated with viral infection. The font size of the pathway labels corresponds to the -log_10_ (Adjusted *p*-value).

Accurately quantifying viral loads in RNA-seq data generated using polyA enrichment protocols is challenging. Therefore, we focused on identifying host-derived biomarkers that reflect the biological impact of viral infection. We analyzed data from two independent studies (GSE198398 and GSE187420) involving two different cell lines (Vero 6 and Calu-3). In the GSE198398 dataset, Vero E6 cells were infected with HCoV-OC43 (OC43), while in GSE187420 dataset, Calu-3 cells were infected with SARS-CoV-2. Differential expression analysis identified 953 and 4,101 DE genes in in the two datasets, respectively (Figures 4a,b). These genes were further subjected to KEGG pathway enrichment analysis, which revealed enrichment of pathways associated with viral infections, including Influenza A, Herpes simplex virus 1, Hepatitis B, Hepatitis C, and Epstein–Barr virus (Figures 4c,d). From this analysis, we selected a list of 300 DE genes that were consistently active in infected samples compared to controls. These genes were then used as input features for feature selection and classification using a Random Forest (RF) model.

### Biomarker discovery for viral contamination in cell lines

**Figure 5:**
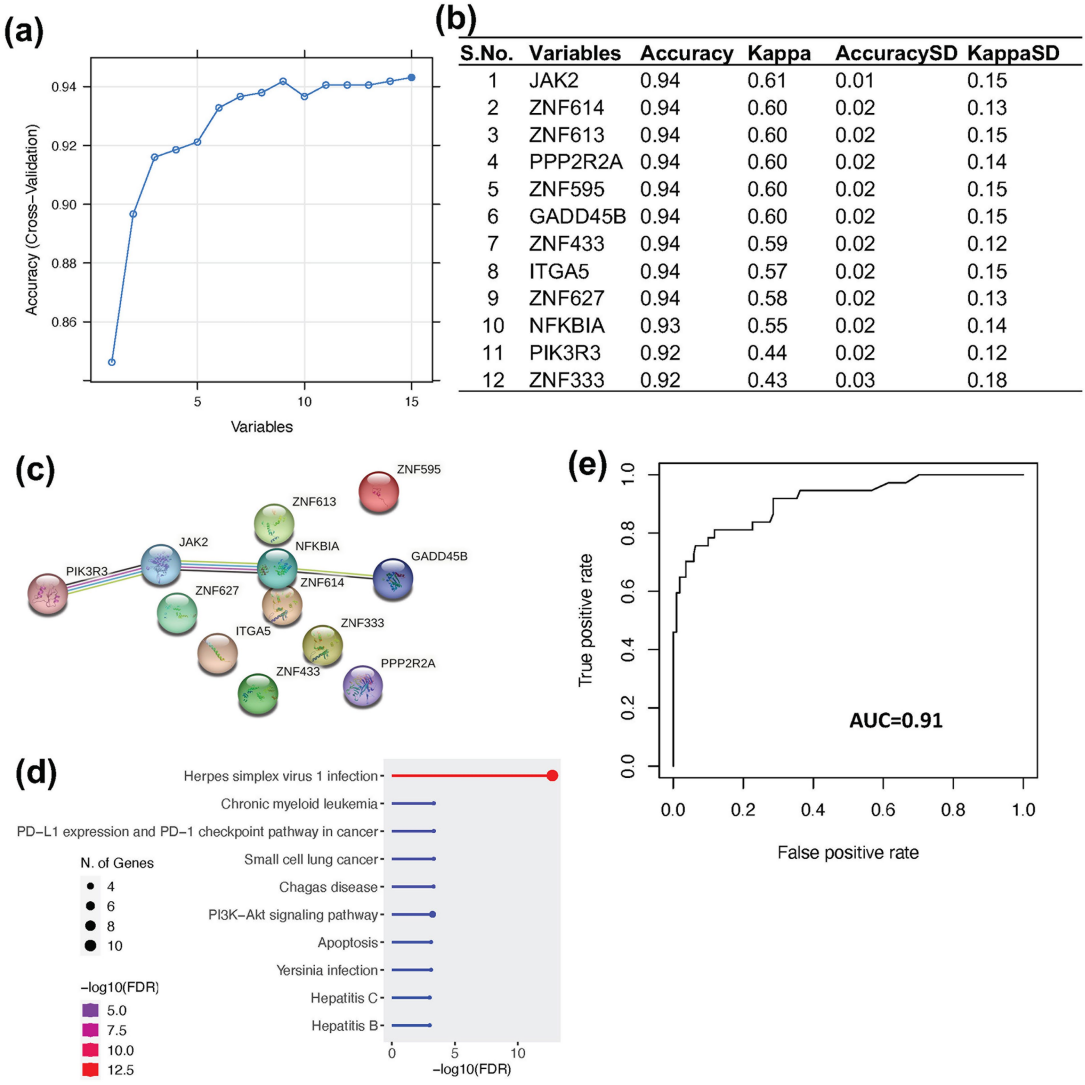
Feature selection and prediction using Random Forest. **(a)** Line plot showing the top 15 features ranked by prediction accuracy in the Random Forest. **(b)** Table listing the top 12 features used for prediction, based on an accuracy threshold of >0.9, resulting in the exclusion of three features. **(c)** Protein–protein interaction network of the 12 selected features generated using STRING database. **(d)** Gene ontology (GO) enrichment of the selected features. **(e)** Model performance on the MCF7 test dataset, achieving an AUC of 0.91, accuracy of 0.93, sensitivity of 0.99, and specificity of 0.60.

After identifying the infected samples in MCF7 cell lines, we categorized them into infected (110 samples) and control (911 samples) groups. Subsequently, we collected the normalized expression matrix of all samples (1,021 samples) from ARCHS4^4^. To establish a gene expression-based biomarker for viral contamination, we applied a RF machine learning approach for feature selection and prediction. The DE genes identified from two independent viral infection datasets, which were enriched in viral infection-related pathways and consistently activity in infected samples, were used as candidate features. Feature selection was performed using the training dataset based on feature importance values generated by the RF model. Top 12 features (*JAK2, ZNF614, ZNF613, PPP2R2A, ZNF595, GADD45B, ZNF433, ITGA5, ZNF627, NFKBIA, PIK3R3,* and *ZNF333*) were selected, each demonstrating classification accuracy greater than 0.92 and a kappa value exceeding 0.44 (Figures 5a,b).

To gain deeper biological insights into these features, we performed protein–protein interaction analysis, gene ontology (GO) enrichment, and KEGG pathway analysis. Among the selected genes, four (PIK3R3, NFKBIA, JAK2, and GADD45B) demonstrated direct interaction with each other (Figure 5c). However, all 12 genes were found to be involved in pathways related to viral infection, cancer, and cell signaling (Figure 5d). These features were subsequently used for predictive modeling. Using RF classification based on these selected features we successfully distinguished infected from control samples, achieving an area under the curve (AUC) of 0.91, an accuracy of 0.93, a sensitivity of 0.99, and a specificity of 0.60 (Figure 5e). In addition, we applied downsampling of the majority class along with 10-fold cross-validation to evaluate the robustness of the model. The model demonstrated a mean accuracy of 0.87 and an average area under the precision-recall curve (AUPRC) of 0.75. These results indicate that the selected features are highly effective for classifying infected and non-infected samples across different cell lines with very high sensitivity. The relatively lower specificity suggests that the biological impact of viral contamination may not uniform across all infected cells.

## Discussion

Cell lines are invaluable tools in biomedical research, providing powerful systems to investigate disease mechanisms and support the development of new therapeutic strategies. However, the use of cell lines is not without challenges, with contamination representing a persistent concern. While bacterial and mycoplasma contamination can often be mitigated through adherence to established laboratory protocols and are relatively straightforward to detect, viral contamination poses a far greater challenge. Viruses are not readily detectable by standard microscopy and often remain undetected without targeted assays.

To address this limitation, we developed ViralCellDetector, a computational pipeline designed to detect viral contaminations directly from RNA-seq data. ViralCellDetector accepts raw sequencing reads as input, systematically filters host-mapped reads, and identifies potential viral sequences by mapping unmapped reads to a comprehensive viral genome database. The pipeline outputs a list of candidate viruses, offering a practical and scalable approach to screen for viral contamination across diverse cell lines and experimental datasets. Additionally, comparison of ViralCellDetector with other tools revealed its better efficacy and faster processing. Building on ViralCellDetector pipeline, we classified a large set of MCF7 cell line experiments into infected and non-infected categories. To further explore the host response to viral contaminations, we identified DE genes from two independent datasets involving two different cell lines and viral infections. The DE genes associated with viral infection were subsequently used in a machine learning approach to identify a gene expression-based biomarker capable of distinguishing infected and non-infected samples. Through this approach, we identified a set of 12 genes as biomarkers that consistently differentiated infected samples.

In our comprehensive analysis of MCF7 cell lines, we detected contamination with three DNA viruses and three RNA viruses. The detected RNA viruses included Encephalomyocarditis virus (EMCV), a member of the Picornaviridae family associated with encephalitis and myocarditis in various mammalian species (Griffin, 2011), and Hepatitis virus, known for its etiological role in hepatocellular carcinoma (Liu, 2020); Among the DNA viruses, we identified *Eptesicus fuscus* gammaherpesvirus (EfGHV), a gammaherpesvirus primarily infecting bat species (Subudhi et al., 2018); BeAn 58,058 virus, a poxvirus isolated from Oryzomys rodents (Wanzeller et al., 2017); Human endogenous retrovirus K113 (HERV-K113), which represents an integrated component of the human genome with potential transcriptional activity under certain conditions; and HIV-1, a well-characterized human retrovirus. Importantly, several of these viruses, such as EMCV, HIV-1, and HERV-K113, are frequently used as experimental models in virology and molecular biology laboratories, which may increase the risk of unintentional carryover into unrelated cell culture systems. However, it is important to note that some of these experiments may have intentionally introduced viral infections as part of their experimental design, and thus not all detected contaminations necessarily reflect unintended viral contamination. Additionally, not all the samples with viral contamination will have impact on host gene expression, and detection of those viruses revealed the virus sequence load in the samples rather any downstream biological effect on the host cells.

Furthermore, the DE analysis in two independent datasets identified a set of genes consistently upregulated in infected samples and associated with viral infection pathways. These genes provided a valuable foundation for feature selection in our machine learning approach. Using this strategy, we identified a list of 12 distinct genes that serve as potential biomarkers capable of accurately classifying infected and non-infected cell lines. Interestingly, these genes are involved in multiple pathways related to viral infection, cellular signaling, and cancer, highlighting their functional relevance in viral entry, replication, and pathogenesis. Several of these genes have already been reported to support viral contamination in cells (Shen et al., 2014; Liu et al., 2017; Talledo et al., 2012). Their strong association with viral infection and key regulatory pathways likely contributes to their high predictive performance. This biomarker offers a valuable tool for the detection of potential viral contamination in cell line experiments, enhancing quality control and enabling researchers to identify unrecognized viral contaminations that may otherwise confound experimental outcomes.

One limitation of the present study is that biomarker discovery was performed using the MCF7 cell line, for which labeled datasets with confirmed viral contamination are not publicly available. To address this, we downloaded and processed fastq files to label over 1,000 MCF7 samples. Additionally, we implemented 10-fold cross-validation and a downsampling approach to assess the robustness of our identified biomarkers. Another limitation is the use of stringent criteria in our pipeline for detecting viral contamination specifically, requiring >20% of reads to be mapped with >50% viral genome coverage. While this enhances the reliability of detection, it may lead to the omission of low-level viral nucleic acids, which may nonetheless have minimal biological impact on the host. Moreover, we validated the tool using simulated datasets and found that even with as little as 10% viral reads, ViralCellDetector was able to detect the viruses with high sensitivity, supporting the effectiveness of our approach.

Future research directions should be considered to further strengthen this work. Our exploratory study revealed a surprisingly widespread occurrence of viral contamination in commonly used cell lines. Further investigations are warranted to elucidate the underlying causes of this phenomenon, which may include laboratory handling practices, viral persistence, or cross-contamination. Such insights will be critical for the scientific community to improve experimental rigor and minimize the risk of unintended viral contamination in future research.

In conclusion, we have developed a computational pipeline and validated the reliability of this tool using the simulated data and compared with identified a biomarker panel for the detection of viral contamination in cell lines using readily available RNA-seq data. This approach offers researchers a valuable tool to investigate unexpected experimental outcomes and implement appropriate corrective measures to ensure data integrity and reproducibility. Incorporating such screening strategies should be considered an essential component of best laboratory practice guidelines.

## Data Availability

The source code of ViralCellDetector is freely available at https://github.com/Bin-Chen-Lab/ViralCellDetector. The data used in this study are public datasets. Those data can be accessible from Gene Omnibus Expression (https://www.ncbi.nlm.nih.gov/geo/browse/?view=series) or Archs4 (https://maayanlab.cloud/archs4/data.html).

## Footnotes

^1^ https://ftp.ncbi.nlm.nih.gov/refseq/release/viral/

^2^ https://www.ncbi.nlm.nih.gov/geo/browse/?view=series

^3^ https://github.com/ncbi/sra-tools/wiki/01.-Downloading-SRA-Toolkit

^4^ https://maayanlab.cloud/archs4/data.html
