# Supplementary material for: Detection of viral contamination in cell lines using ViralCellDetector
Source: Front Microbiol. 2025 Aug 13;16:1595180. doi: 10.3389/fmicb.2025.1595180 (PMC12380829; doi:10.3389/fmicb.2025.1595180)
Supplement: Supplementary file 1 [file Data_Sheet_1.PDF]

Table S1. Percentage of raw sequencing reads that were not mapped to the human reference transcriptome.

Sample	Condition	Unmapped reads on host reference transcriptome (%)
GSM5668291	CT	8.17
GSM5668292	CT	9.07
GSM5668293	CT	7.94
GSM5668294	Infected	89.72
GSM5668295	Infected	88.19
GSM5668296	Infected	88.12

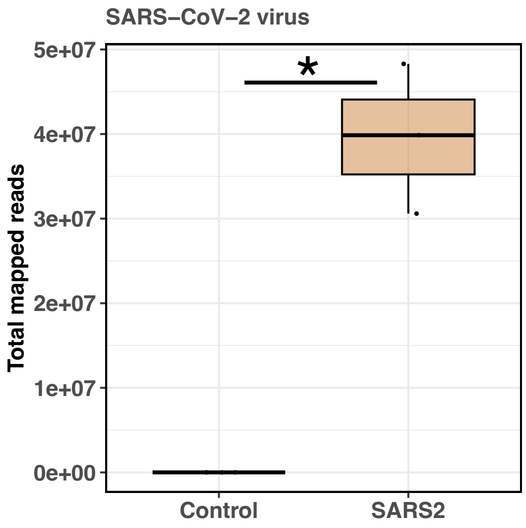

Fig. S1. Total mapped reads on the SARS-CoV-2 genome using the VirusCelldetector package. The infected samples showed millions of read mapped on the SARS-CoV-2 genome, whereas control sample has less than 2000 reads belongs to SARS-CoV-2 genome.

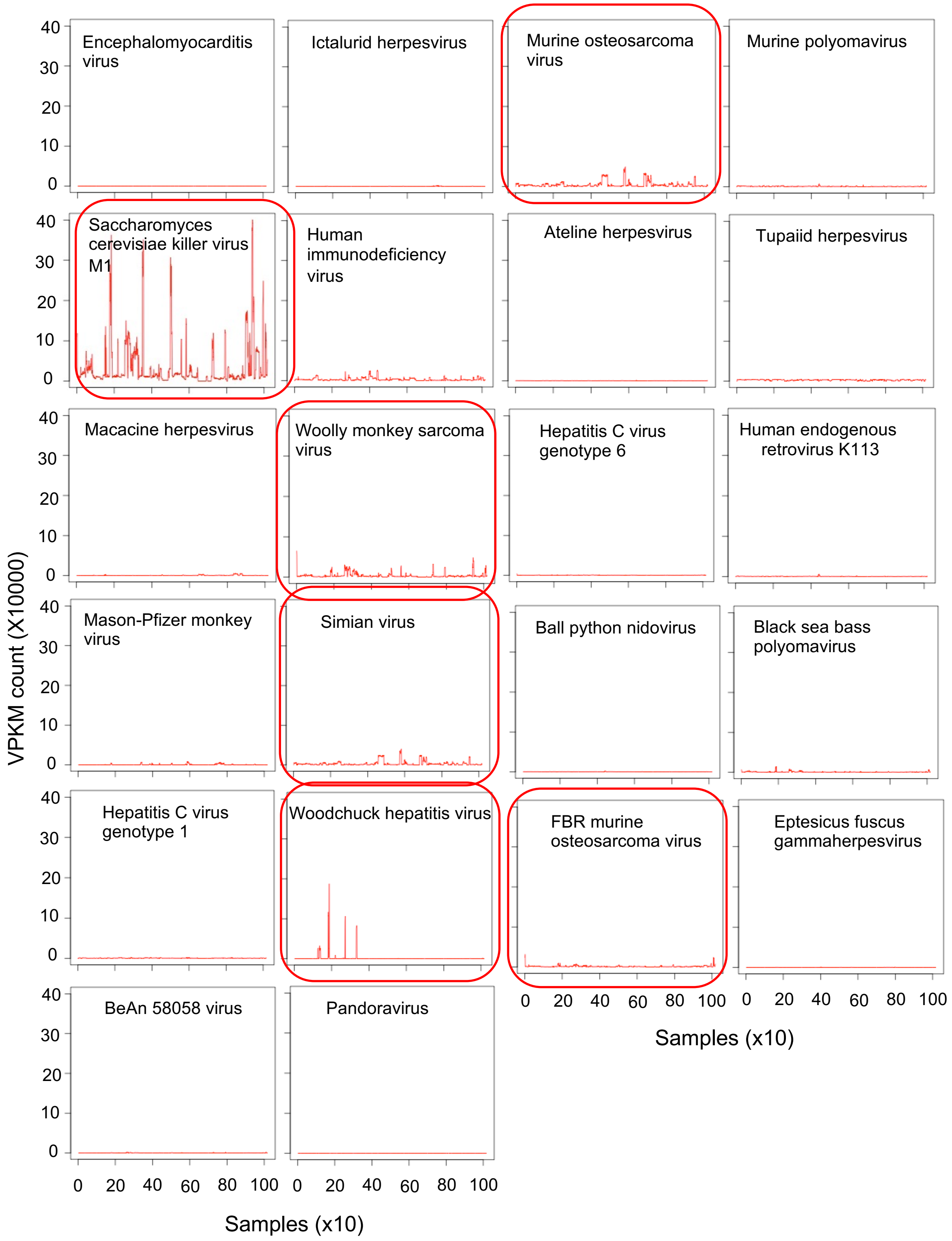

Fig. S2. Virus per kb per million reads (VPKM) count of each virus in different experiments in MCF7 cell lines. Only six viruses showed the difference in their VPKM count in different experiments in MCF7 cells.

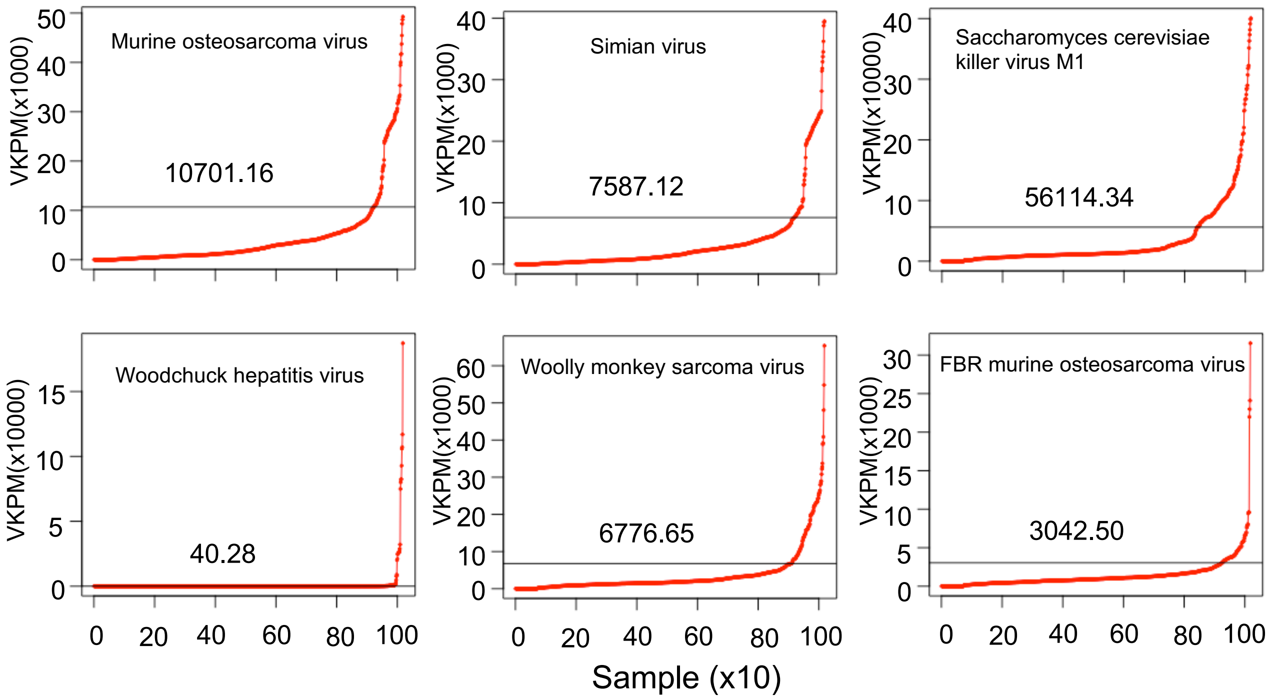

Fig. S3. Identification of samples with very high viral expression. We used the upper quartile to filter the samples with very high viral count.

Table S2 list of samples used for differentially expressed gene analysis in GSE198398.

S.No.	Sample	Condition
1	GSM5946242	CT
2	GSM5946243	CT
3	GSM5946244	CT
4	GSM5946245	infected
5	GSM5946246	infected
6	GSM5946247	infected

Table S3 list of samples used for differentially expressed gene analysis in GSE187420.

S.No.	Sample	Condition
1	GSM5668291	CT
2	GSM5668292	CT
3	GSM5668293	CT
4	GSM5668294	infected
5	GSM5668295	infected
6	GSM5668296	infected
